# Development of a Conditional *Mesd (Mesoderm Development)* Allele for Functional Analysis of the Low-Density Lipoprotein Receptor-Related Family in Defined Tissues

**DOI:** 10.1371/journal.pone.0075782

**Published:** 2013-10-04

**Authors:** Andrew V. Taibi, Janet K. Lighthouse, Richard C. Grady, Kenneth R. Shroyer, Bernadette C. Holdener

**Affiliations:** 1 Department of Biochemistry and Cell Biology, Stony Brook University, Stony Brook, New York, United States of America; 2 Department of Pathology, Stony Brook University, Stony Brook, New York, United States of America; Montana State University, United States of America

## Abstract

The Low-density lipoprotein receptor-Related Protein (LRP) family members are essential for diverse processes ranging from the regulation of gastrulation to the modulation of lipid homeostasis. Receptors in this family bind and internalize a diverse array of ligands in the extracellular matrix (ECM). As a consequence, LRPs regulate a wide variety of cellular functions including, but not limited to lipid metabolism, membrane composition, cell motility, and cell signaling. Not surprisingly, mutations in single human LRPs are associated with defects in cholesterol metabolism and development of atherosclerosis, abnormalities in bone density, or aberrant eye vasculature, and may be a contributing factor in development of Alzheimer’s disease. Often, members of this diverse family of receptors perform overlapping roles in the same tissues, complicating the analysis of their function through conventional targeted mutagenesis. Here, we describe development of a mouse *Mesd (Mesoderm Development)* conditional knockout allele, and demonstrate that ubiquitous deletion of *Mesd* using Cre-recombinase blocks gastrulation, as observed in the traditional knockout and albino-deletion phenotypes. This conditional allele will serve as an excellent tool for future characterization of the cumulative contribution of LRP members in defined tissues.

## Introduction

MESD is an endoplasmic reticulum (ER) chaperone whose function is specialized for folding the β-propeller/Epidermal Growth Factor (EGF) module characteristically found in the extracellular domains of the Low-density lipoprotein receptor-Related Protein (LRP) family [1]–[3]. Ten mammalian LRPs contain the β-propeller/EGF module: Low Density Lipoprotein Receptor (LDLR), Very Low Density Lipoprotein Receptor (VLDLR), LDLR-Related Protein 1 and 1b (LRP1 and LRP1b), Megalin (LRP2), Apolipoprotein E Receptor 2 (ApoER2), LDLR-Related Protein 4 (LRP4 or Megf7), LDLR-Related Protein 5 and 6 (LRP5 and 6), and Sorting receptor related (SorLA) [4]–[6]. Because of their diverse roles in cell signaling and endocytosis, mutations in LRPs lead to phenotypes ranging from developmental defects to elevated serum lipids in the adult [7]–[9]. Often multiple LRPs perform overlapping roles in a given tissue, complicating functional analysis [7], [8].

Because MESD is required for localization of the β-propeller/EGF module characteristic of LRPs, tissue specific disruption of *Mesd* should simultaneously disrupt all LRPs, and therefore provides a valuable tool for understanding the collective contribution these receptors make to tissue differentiation and function. To begin to address the role of LRPs in defined cells and tissues, we developed a conditional *Mesd* allele, and demonstrate that ubiquitous deletion of *Mesd* using a PGK promoter driven Cre-recombinase recapitulates the conventional knockout and albino-deletion phenotypes. In addition, using adenovirus delivered Cre-recombinase (adCre) we demonstrate that deletion of *Mesd* in hepatocytes can be achieved in adult cells. However, given the variable efficiency of infection and recombination achieved through delivery of adCre, we recommend that future studies evaluating LRP function in hepatocytes use inherited tissue specific Cre-recombinase transgenes.

## Methods

### Ethics Statement

All animal work was conducted according to relevant national and international guidelines. Stony Brook University operates under Assurance #A3011-01, approved by the NIH Office of Laboratory Animal Welfare (OLAW). The animal studies were approved by the Stony Brook University Institutional Animal Care and Use and Committee (IACUC, 267267) which follow all the guidance set forth in: Public Health Service Policy on Humane Care and Use of Laboratory Animals distributed by Office of Laboratory Animal Welfare, NIH; Animal Welfare Act and Animal Welfare Regulations distributed by United States Department of Agriculture; and Guide for the Care and Use of Laboratory Animals distributed by the National Research Council. Stony Brook University animal facilities are accredited with AAALAC International (Association for the Assessment and Accreditation of Laboratory Animal Care International). Recombinant DNA use was approved by the Stony Brook University Institutional Biosafety Committee (IBC, 267264).

### Mouse Strains and Generation of the *Mesd* Conditional Knockout

Mice heterozygous for the Mesd albino deletion, *Del(7)Tyr^c-3YPSD^*/*Tyr^c-ch^* (*Mesd-3YPSD*), were maintained in a closed colony by crossing to (*Tyr^C^/Tyr^c-ch^*) [10]. The conventional Mesd knockout, Mesdc2^tm1bch^ (*Mesd-KO*), was generated in 129 ES cells, and mice are now maintained as a congenic stock by back-crossing to *C57BL/6J* [2]. *Mesdc2^tm1bch^* is available from The Jackson Laboratory, stock number: 013577.

The *Mesd* conditional knockout allele (C57BL/6-*Mesdc2^tm2bch^* (*Mesd-floxed*)) was generated by *Ozgene*. The targeting construct was electroporated into the C57BL/6 embryonic stem cell (ES) cell line, Bruce4. Three homologous recombinant cell lines were identified among the approximately 480 cell lines screened. Homologous recombinant ES cells were injected into blastocysts from C57BL/6-albino mice, which were congenic for the Tyr<c-Brd> mutation on the C57BL/6Ncr genetic background. Chimeras were mated to C57BL/6-albino, and resulting heterozygous progeny were either backcrossed to C56BL/6J or crossed to PGK-Cre-recombinase expressing mice obtained from *Ozgene* (C57BL/6J background).

Backcross progeny heterozygotes were intercrossed to generate *Mesd-Floxed* homozygotes. Homozygous *Mesd-Floxed* mice are viable and fertile and were maintained by intercrossing. The level of MESD expressed in these animals was not determined and the *Neo* cassette was not removed by Flp-mediated recombination. C57BL/6-*Mesdc2^tm2bch^* (*Mesd-floxed*) will be deposited and available at the Mouse Mutant Regional Resource Center (MMRC), MMRRC: 036939.

### Genotyping

**Figure 1:**
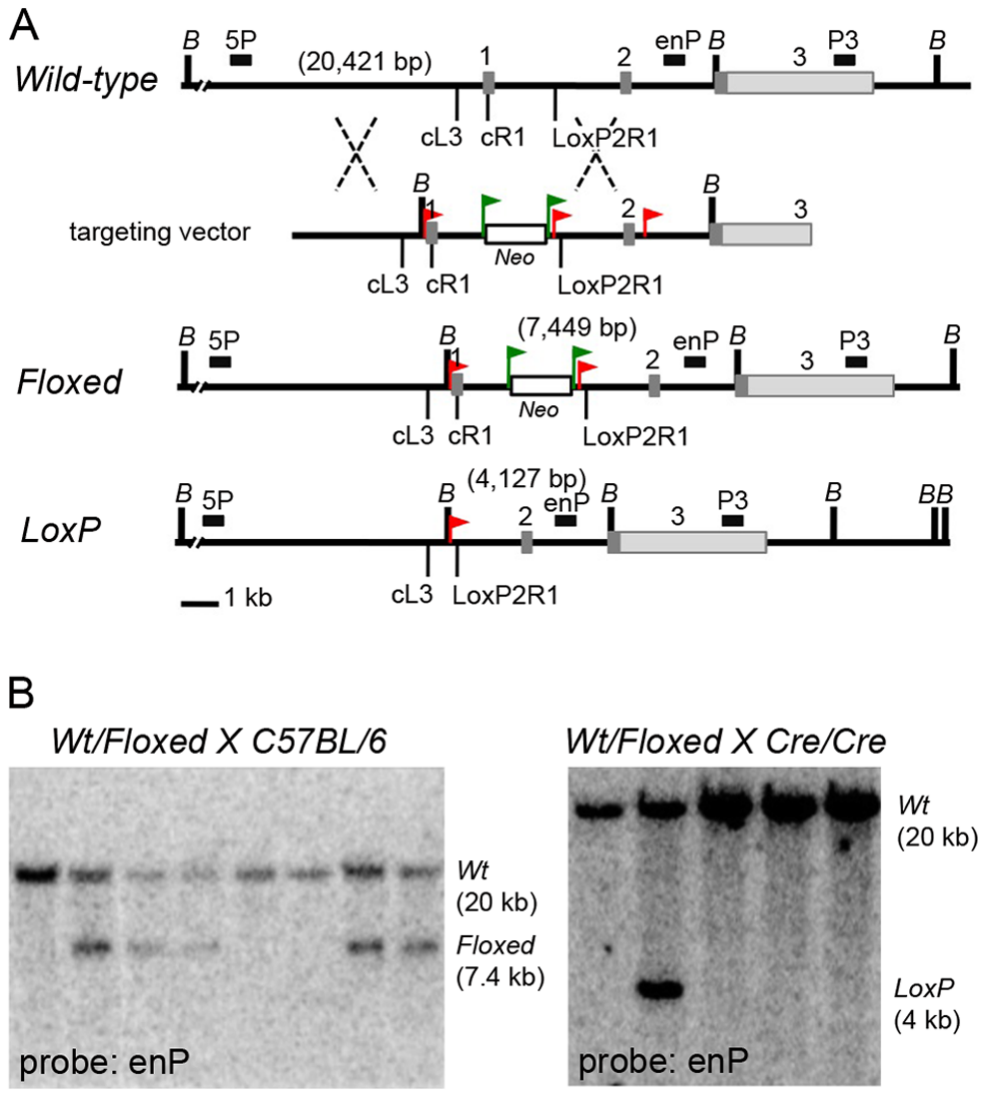
The *Mesd* conditional allele. (**A**) Comparison of the *Mesd Wild-type, Floxed,* and *LoxP Mesd* alleles and targeting vector. The *Wild-type Mesd* allele (top map) has three exons (1–3) that are designated by grey rectangles. The first exon encodes the signal peptide that directs the MESD protein into the ER as well as the N-terminal helical region essential for maturation of LRPs [1], [3]. The 3′ untranslated portion of the third exon is indicated by light grey. In the *Mesd* targeting vector, we introduced the *Neomycin resistance* gene (*Neo*) (white rectangle) flanked by Flp-recombinase sites (green triangles) into the *Mesd* first intron. In addition, we introduced three LoxP sites (red triangles) flanking the first and second exons and the *Neo* gene.The targeting construct was introduced into C57BL/6 embryonic stem cells (*Ozgene*), and cell lines heterozygous for the *Floxed* allele (middle map) were identified by Southern analysis using the 5P, enP, and P3 probes, whose positions are indicated by black rectangles (described in Materials and Methods and Table 1). Based on Southern analysis using these probes, we determined that only the first two LoxP sites were incorporated into ES cells; the recombination sites are indicated by dashed lines. Expression of Cre-recombinase will trigger recombination between LoxP sites to generate the *LoxP* allele (bottom map). (**B**) Heterozygous *Floxed* mice were mated to C57BL/6 mice (left) for strain propagation or to animals homozygous for a ubiquitously expressed Cre-recombinase (right) to generate the recombined *LoxP* (knockout) allele. Progeny were genotyped by Southern analysis of *BamH*I digested DNA using the enP probe. This probe clearly distinguished the *Wt* (20 kb) fragment from the *Floxed* (7.4 kb) or *LoxP* (4 kb) fragments.

Genotyping of *Mesd* albino deletion was determined by coat color; heterozygous deletion carriers, *Del(7)Tyr^c-3YPSD^/Tyr^c-ch^*, are light grey in appearance. Genotyping of *Mesd* conventional knockout, *Mesdc2^tm1bch^*, was performed by PCR as previously described [2]. Genotyping of the *Mesd* conditional allele, *Mesdc2^tm2bch^* (*Mesd-Floxed*), and resulting recombined *Mesd-LoxP* allele (single *LoxP* site remaining after cre-mediated recombination) was initially determined by Southern analysis and subsequently by polymerase chain reaction (PCR). For Southern analysis of the *Mesd* conditional allele, tail DNA was digested with *Bam*HI and probed with the 5P, P3, or enP probes (refer to Figure 1). 5P, P3, and enP probes were generated by PCR amplification using primers described in Table 1. Primer sets and product sizes used to distinguish the *Mesd*-*Wild-type, Floxed*, or *LoxP* alleles, and *Cre-recombinase* using PCR are also described in Table 1. Multiplex PCR including primers: *Mesd-CommonL3, Mesd-CommonR1,* and *Mesd-LoxP2R1* was performed using *Invitrogen Platinum ® Taq* DNA polymerase high fidelity, 1× high fidelity buffer supplemented with 1.4 mM MgSO4 and 0.25% dimethyl sulfoxide, and cycling as follows: 30 seconds at 95°C; then 30 cycles of 30 seconds at 95°C, 30 seconds at 55°C, and 30 seconds at 68°C; followed by 5 minutes at 68°C and hold at 15°C.

**Table 1:** Primers for genotyping.

Primer Name	Primer sequence 5′–3′	Size (bp)	Probe/Allele
P1186_11	ATGGCATTGCTTAGTCTCTTTGAAG	607	EnP probe
P1186_12	GACAGAAATCCTAACCTCCAAGGTC		
P431_01	TTCAGGGTCTGTAGTTGGTGATTGT	459	5P probe
P431_02	GGCGGCTATCCAGGTTTTTACC		
P431_03	GCTAACACTGCCATCATTGCACT	474	P3 probe
P431_04	TCTGATCTGGAACTTCTGATCTTGC		
Mesd-CommonL3	CAAGTGGTGAAAGGCTCGAT	508	*Wild-type* allele
Mesd-CommonR1	GTTGTAATCGCGGATGTCCT	589	*Floxed* allele
Mesd-CommonL3	CAAGTGGTGAAAGGCTCGAT	1,822	*Wild-type* allele
Mesd-LoxP2R1	CCTACCCCCTTGTTCTGACA	489	*LoxP* allele
Cre-L1	GCATTACCGGTCGATGCAACGAGTGATGAG	403	*Cre-recombinase*
Cre-L2	GAGTGAACGAACCTGGTCGAAATCAGTGCG		

### Assessing Cre-recombinase Mediated Deletion of *Mesd* in Liver

Replication defective adenovirus expressing Cre-recombinase-GFP (adCreGFP) was obtained from the Stony Brook University Stem Cell Facility, and 2×10^9^ pfu in 100 µl PBS was introduced into the tail-vein of four *Mesd-Floxed* homozygous mice. GFP expression in two animals was evaluated 48 hours after infection and DNA isolated. Photography of whole livers was performed using Discovery V.20 Stereo Dissecting Microscope (Zeiss) with a mono 3.5× objective and AxioCam HRc camera. Photographs were analyzed using AxioVision v4.8 software. To examine the effects of *Mesd* deletion on hepatocyte function, two weeks after infection with adCreGFP, animals were placed on a *Research Diets, Inc.* high fat diet with regular casein and 1.25% added cholesterol (Clinton/Cybulsky D12108C) for eight weeks; animal weight was measured 4 to 5 times each week. During this time, animals were allowed free access to food and water. Liver samples were collected for histology (not shown), and DNA was then extracted from liver and brain tissue. PCR-based assessment of recombination was as described for genotyping, except that the total number of cycles was reduced to 25.

## Results and Discussion

### Cre-recombinase Mediated Deletion of the *Mesd* Conditional Knockout Allele Recapitulates the *Mesd* Knockout and Albino-deletion Phenotypes

To facilitate analysis of *Mesd* function in defined tissues and at specific time points throughout development and in the adult, we generated mice carrying a conditional *Mesd* knockout allele using the Cre-LoxP system [11]. To generate the *Mesd-Floxed* allele, we introduced the *neomycin resistance (Neo)* gene into the *Mesd* first intron and *LoxP* sites in the *Mesd* promoter and downstream of the *Neo* cassette (Figure 1A). We identified targeted embryonic stem cells by Southern hybridization using the enP (Figure 1A and B, Table 1), 5P, and P3 hybridization probes (Figure 1A, data not shown, Table 1). The enP probe detected a 20 kb Bam*HI* fragment in *Wild-type* mice and both 20 kb and 7.4 kb fragments in heterozygous carriers of the conditional allele (*Mesd-Floxed*) (Figure 1B left panel). After PGK::Cre-recombinase mediated deletion of exon 1, the enP probe detected the 4.0 kb *Mesd-LoxP* allele in DNA isolated from tail biopsy.

**Figure 2:**
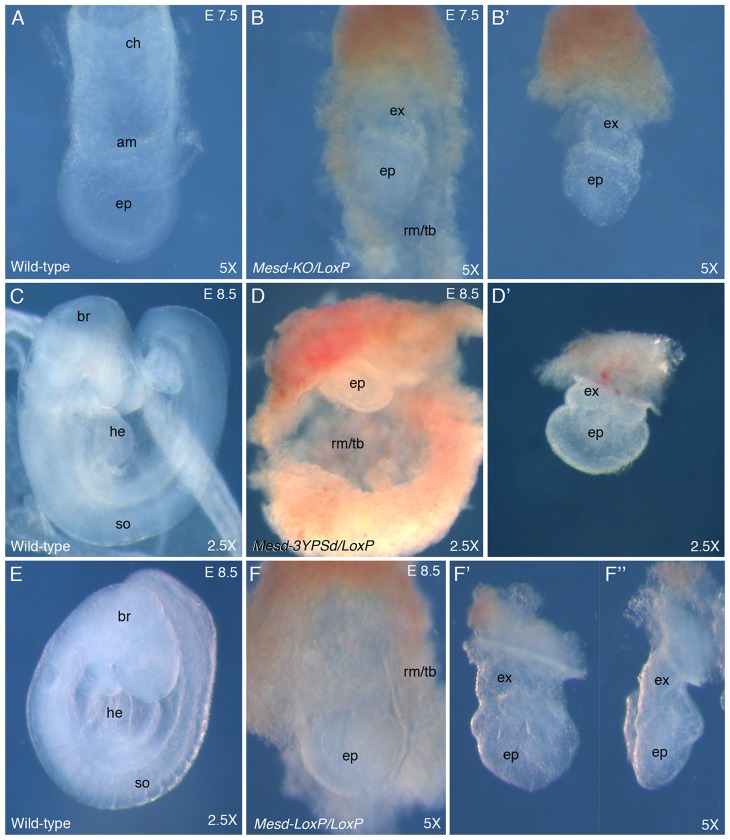
The *Mesd-LoxP* allele does not complement the *Mesd* knockout and albino deletion alleles. Animals heterozygous for the Cre-recombined *Mesd* (*Mesd-LoxP*) allele were mated to animals heterozygous for the *Mesd* conventional knockout (*Mesd-KO*) (A–B), the albino deletion (*Mesd-3YPSD*) deletion (C–D), or the *Mesd-LoxP* allele (E–F). Embryos were dissected at embryonic day (E) 7.5 or E 8.5 and photographed at the indicated magnifications (5× or 2.5×). (**A**) At E 7.5, gastrulation was near completion in wild-type embryos and the epiblast (ep), amnion (am) and chorion (ch) were clearly visible. (**B, B’**) In contrast, in the *Mesd* mutant littermate gastrulation was blocked, the extra-embryonic ectoderm (ex) and epiblast (ep) were organized as epithelia, and Reichert’s membrane and trophoblast (rm/tb) were expanded in comparison to the epiblast. In panel B’, Reichert’s membrane was removed to facilitate better visualization of the *Mesd* egg-cylinder. (**C** and **E**) By E 8.5 the size of the wild-type embryo had increased considerably, and the brain (br), heart (he), and somites (so) were clearly visible. (**D–F’’**) In contrast, the mutant littermates had only doubled in size, and the epiblast (ep) remained undifferentiated, and Reichert’s membrane and trophoblast (rm/tb) were greatly expanded; this layer was removed in panel D’ and F’. The embryo shown in panel F’ was rotated 90 degrees (**F’’**) to allow visualization of the shortened anterior/posterior axis and characteristic indentation on the left side of the pictured *Mesd* mutant. Previously, we demonstrated that the indentation was coincident with expression of Hex [1].

Since the signal peptide directing the MESD protein into the endoplasmic reticulum (ER) and the N-terminal α-helical region essential for folding the LRP β-propeller/EGF motif are encoded by exon 1 [3], we predicted that Cre-recombinase mediated deletion of *Mesd* exon 1 (*Mesd-LoxP*) would result in the loss of MESD function. To test this hypothesis, we verified that the *Mesd-LoxP* allele recapitulates the *Mesd-3YPD* albino deletion and *Mesd-KO* knockout phenotypes (Figure 2). *Mesd-LoxP* heterozygotes were crossed to *Mesd-KO, Mesd-3YPSD,* or *Mesd-LoxP* heterozygotes, and embryo phenotypes were examined at E 7.5 and E 8.5. At E 7.5 wild-type embryos had undergone gastrulation, and extra-embryonic membranes (amnion and chorion) were visible (Figure 2A). By E 8.5 the wild-type embryos began organogenesis, with brain, heart, and somites visible (Figure 2C and E). In contrast, *Mesd-LoxP/Mesd-KO* (Figure 2B) and *Mesd-LoxP/Mesd-3YPSD* compound heterozygotes (Figure 2D), and *Mesd-LoxP* homozygotes (Figure 2F) failed to differentiate mesoderm or undergo gastrulation. Although the growth of Reichert’s membrane and trophoblast appeared unimpaired in *Mesd* mutants (Figure 2D), the epiblast was considerably smaller than normal littermates at E 7.5 and E 8.5 (Figure 2B, D, and F). Between E 7.5 and E 8.5, the size of the *Mesd* mutant epiblast did increase (compare Figure 2B’ and F’). However unlike normal littermates, the *Mesd* mutants failed to orient the anterior/posterior axis with the long axis of the epiblast; the anterior side of the epiblast had a characteristic indentation (compare Figure 2F’ and F’’). The similarity of *Mesd-LoxP* homozygotes with the previously described homozygous albino deletion and knockout phenotypes [2], as well as the failure of the *Mesd-LoxP* allele to complement the *Mesd-KO* and *Mesd-3YPSD* null alleles, provided strong evidence that PGK-Cre-recombinase mediated deletion of *Mesd* exon 1 (*Mesd-LoxP*) generated a null allele and was well-suited for analysis of MESD/LRP function in defined tissues.

### Assessment of Cre-recombinase Mediated *Mesd* Deletion in Adult Liver

**Figure 3:**
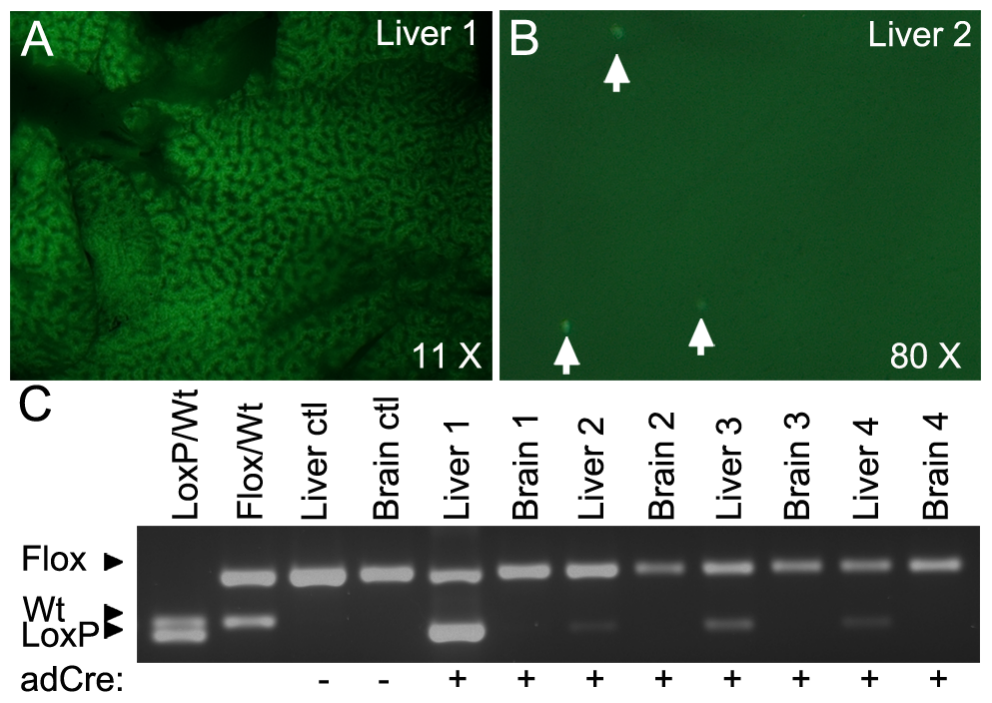
Assessment of *Mesd* recombination in liver in response to adCreGFP infection. (**A–B**) GFP fluorescence in *Mesd-Floxed* homozygous livers 48 hours post adCre-GFP injection. (**A**) Liver 1 was photographed at 17 ms exposure on a dissecting microscope under 535nm UV light at 11.0×magnification. (**B**) In contrast, liver 2 showed fluorescence in only sporadic cells, and was photographed at 80×magnification and at a 432 ms exposure, to capture the few fluorescing cells. (**C**) PCR amplification to assess adCre mediated excision of *Mesd-Floxed.* (Lanes 1–2) *Mesd-LoxP/Mesd-Wild-type* (LoxP/Wt) and *Mesd-Floxed/Mesd-Wild-type* (Flox/Wt) tail DNA preparations were used for size reference. (Lanes 3–4) genomic DNA prepared from livers and brains isolated from non-infected animal. (Lanes 5–12) Genomic DNA prepared from liver and brain isolated from adCreGFP infected animals 48 hours post infection (animal 1 & 2) and ten weeks post infection (animals 3 & 4).

In the liver, several LRPs including LDLR, LRP1, and LRP5 regulate lipid and glucose homeostasis. Conditional deletion of *Mesd* in hepatocytes could provide an effective method for assessing the collective contribution of LRPs to this process. Previously, adenoviral delivery of Cre-recombinase was shown to preferentially infect hepatocytes and induce recombination with close to 100% efficiency in these cells [12]. However, the level of recombination that can be achieved using adenovirus delivery depends on experimental variables such as the efficiency of the injection, the infection of hepatocytes, and the recombination at a particular locus. To determine whether this would be a suitable method for analysis of MESD function in adult hepatocytes, we infected four *Mesd-Floxed* homozygotes with replication defective adenovirus-Cre-GFP (adCreGFP). Forty-eight hours after adenovirus injection we evaluated the efficiency of adenovirus infection and recombination in two animals using GFP expression and PCR. GFP was expressed throughout the hepatocytes of Animal 1 (Figure 3A), but was only detected in sporadic hepatocytes of Animal 2 (Figure 3B), suggesting inefficient adenoviral infection. Neither mouse showed fluorescence in other organs, confirming the specificity of adCreGFP for hepatocytes [12], [13]. The level of observed GFP fluorescence (Figure 3 A and B) was consistent with the level of Cre-mediated recombination suggested by PCR amplification of the LoxP allele (Figure 3C).

Two weeks post adenovirus-Cre-GFP infection, two remaining infected animals and two non-infected floxed homozygous animals were placed on high fat diet for 8 weeks. At the end of this period, only low levels of *Mesd* recombination were detected in two remaining livers 10 weeks post infection; no differences were observed in liver pathology on high fat diet compared to non-infected floxed animals (not shown), suggesting that the low-level recombination likely resulted from initial inefficient hepatocyte infection rather than death of *Mesd*-deficient cells.

## Conclusions

Ubiquitous deletion of *Mesd* using PGK::Cre-recombinase, recapitulated the *Mesd-3YPSD* deletion and *Mesd-KO* phenotypes, verifying that deletion of *Mesd* exon 1 resulted in a null allele. Furthermore, high levels of *Mesd* excision in the animal with efficient adenovirus-Cre-GFP infection, confirms that the *Mesd* conditional allele efficiently recombines in hepatocytes, and will be effective for analysis of tissue specific knockout phenotypes using suitable cre-expressing mouse strains. However, variations in hepatocyte infection frequency and recombination frequency detected by PCR in four animals provided evidence that reliable assessment of MESD function in hepatocytes will require genetic introduction of hepatocyte driven Cre-recombinase.

With no overt *Mesd-Floxed* homozygous defects, the *Mesd* conditional line can be used in tissue specific and temporally controlled loss-of-function experiments in embryonic and adult tissues. *Mesd* mutant embryos continue to express pluripotency markers *Oct4, Nanog,* and *Sox2* [1], [2], [10], and mutant embryonic stem cells do not differentiate in teratomas [10]. This observation suggests that LRP function may also be essential for the progression of the epiblast from a ground state to a state competent to respond to differentiation signals. Conditional deletion of *Mesd* epiblast or primitive endoderm will determine whether this progression is modulated by LRP function in the epiblast or extra-embryonic tissues. Importantly, loss of MESD provides a mechanism to simultaneously assess the combined contribution of LRPs to tissue function and embryogenesis.
